# Socio-economic disparities in the dispensation of antibiotics in Sweden 2016–2017: An intersectional analysis of individual heterogeneity and discriminatory accuracy

**DOI:** 10.1177/1403494820981496

**Published:** 2021-01-18

**Authors:** Maria Wemrell, Cecilia Lenander, Kristofer Hansson, Raquel Vicente Perez, Katarina Hedin, Juan Merlo

**Affiliations:** 1 Unit for Social Epidemiology, Department of Clinical Sciences in Malmö, Lund University, Sweden; 2 Department of Gender Studies, Lund University, Sweden; 3 Family Medicine, Department of Clinical Sciences in Malmö, Lund University, Sweden; 4 Department of Social Work, Malmö University, Sweden; 5 Futurum, Region Jönköping County, and Department of Health, Medicine and Caring Sciences, Linköping University, Sweden; 6 Center for Primary Health Care Research, Region Skåne, Sweden

**Keywords:** Antibiotic medication, antimicrobial resistance, socio-economic disparities, Sweden, intersectionality

## Abstract

*Aims:* Antimicrobial resistance presents an increasingly serious threat to global public health, which is directly related to how antibiotic medication is used in society. Actions aimed towards the optimised use of antibiotics should be implemented on equal terms and according to the needs of the population. Previous research results on differences in antibiotic use between socio-economic and demographic groups in Sweden are not entirely coherent, and have typically focused on the effects of singular socio-economic variables. Using an intersectional approach, this study provides a more precise analysis of how the dispensation of antibiotic medication was distributed across socio-economic and demographic groups in Sweden in 2016–2017. *Methods:* Using register data from a nationwide cohort and adopting an intersectional analysis of individual heterogeneity and discriminatory accuracy, we map the dispensation of antibiotics according to age, sex, country of birth and income. *Results:* While women and high-income earners had the highest antibiotic dispensation prevalence, no large differences in the dispensation of antibiotics were identified between socio-economic groups. ***Conclusions:* Public-health interventions aiming to support the reduced and optimised use of antibiotics should be directed towards the whole Swedish population rather than towards specific groups. Correspondingly, an increased focus on socio-economic or demographic factors is not warranted in interventions aimed at improving antibiotic prescription patterns among medical practitioners.**

## Introduction

Antimicrobial resistance (AMR) presents an increasingly serious threat to global public health, which is directly related to how antibiotic medication is used in society [1]. In Sweden, while the situation is favourable in comparison to many other countries, and the number of annual prescriptions per capita has decreased over recent years [2], actions aiming towards the optimisation of antibiotic use are still warranted.

The reduction and optimisation of antibiotic use should be implemented on equal terms and according to the needs of the population [3]. We know that differences in antibiotic prescription patterns have existed between counties within the EU [4,5], between parts of Sweden [2,6] and between socio-economic and demographic groups [7,8]. Meanwhile, it has been noted that in public debates, responsibility and blame for AMR sometimes tend to be assigned to specific groups in society [9,10].

Research findings on differences in antibiotic use between socio-economic groups in Sweden (e.g. with regard to education [6,7]) are, however, not entirely coherent. Moreover, the study of social determinants of antibiotic use has typically focused on the effects of singular dimensions, such as socio-economic status, sex or race/ethnicity/racialisation. In recent years, however, an intersectional [11] perspective enabling understanding of how such dimensions interweave in the formation of health inequities has been promoted [12–14]. In this study, we operationalised an intersectional approach through the analysis of individual heterogeneity and discriminatory accuracy (AIHDA) [14–16].

### Intersectionality and AIHDA

There are a number of potential contributions of an intersectional AIDHA approach to social epidemiology. The first of these is an increased specificity in the mapping of health inequalities through providing information about the distribution of risk between strata defined by combinations of different demographical and socio-economic dimensions (i.e. variables). Second, intersectional AIHDA yields information about the variability within and overlaps between social strata in relation to the health outcome [16]. This is done through complementing conventional measures of differences between the average risk of the studied groups with assessments of the discriminatory accuracy (DA) of the variables, that is, their capacity to differentiate between individuals with or without the outcome [14,16,17]. This is important in the interest of counteracting simplification or essentialisation of difference between groups, and for avoiding unnecessary stigmatisation of groups with a higher average risk, the latter in potential accordance with culturally informed and power-implicated perceptions of ‘the Other’ [10]. Moreover, an intersectional perspective promotes the direction of focus towards societal structures and dynamics giving rise to health inequalities, rather than the understanding of social categorisations or levels of risk as essential characteristics of individuals or groups [12,14,18]. Thus, intersectional AIHDA, which has been further described elsewhere [14–16], provides an improved instrument for risk assessments and public-health interventions.

### Aim

Against the background of inconsistent research results on differences in antibiotic dispensation between groups, and the importance of such knowledge due to its potential implications for interventions targeted towards prescribers and the public, this study aimed to provide a more precise mapping of how the dispensation of antibiotics is distributed across socio-economic and demographic groups in Sweden.

## Methods

### Study population

This was a register study based on data linking the Register of the Total Swedish Population (TPR) and the Longitudinal Integration Database for Health Insurance and Labour Market Studies (LISA) administered by Statistics Sweden (Statistiska Centralbyrån), with the Swedish Prescribed Drug Register (SPDR) administered by The National Board of Health and Welfare (Socialstyrelsen). The SPDR contains information about all drug dispensations (except from stockpiles in nursing homes and hospital wards) by the Anatomical Therapeutic Chemical (ATC) code, while the LISA database provides demographic and socio-economic information. The record linkage was performed by The National Board of Health and Welfare and Statistics Sweden after revision by their data safety committees. The study was approved by the Regional Ethics Committee (Dnr 2014/856).

Our research database consisted of the Swedish total population of 2010, and this cohort was followed prospectively for the purpose of analysing dispensation of antibiotics over a two-year period: 2016–2017. From the approximately 9.4 million people originally included in the 2010 population, we excluded those who died (*n*=185,751) or emigrated (*n*=75,492) between 2010 and 2017 and those whose country of birth was unknown (*n*=68,575). The final sample consisted of around 8.1 million people. Because the data were based on the 2010 population, all people included in the 2017 cohort were at least seven years old.

### Variables

Our outcome variable was antibiotic dispensation (ATC codes J01, excluding J01XX05 methenamine) during 2016–2017 (yes vs. no).

The explanatory variables were age, sex, country of birth and income. The age variable was divided into eight groups: 7–14, 15–24, 25–34, 35–44, 45–54, 55–64, 65–74 and ⩾75 years. Sex was coded as male or female. Regarding country of birth, we distinguished between Sweden; Nordic countries excluding Sweden; Europe excluding Nordic countries; the USA, Canada and Australia; and Asia, Africa and Central and South America. We used information on individualised disposable family income for the years 2000, 2005 and 2010 to compute a cumulative measure which was less sensitive to temporary fluctuations in income than single measurements and which mitigated against reverse causality [19]. We used information on absolute income considering the size of the household and the consumption weight of the individuals according to Statistics Sweden. For each of the three years, income levels were categorised into 25 groups (1–25) by quantiles using the complete Swedish population. These groups from the respective three years were summed up, so that each individual received a value between 3 (always in the lowest income group) and 75 (always in the highest income group). We categorised this cumulative income into three groups by tertiles (low, medium or high income). Individuals with missing values on income during 2000 or 2005 (*n*=1002) were assigned the tertile values for the year 2010. No individuals had missing income data for 2010.

Our intersectional variable was constructed though all possible combinations of the mentioned explanatory variables (8×2×5×3), thus forming 240 intersectional strata. We used 45- to 54-year-old men born in Sweden with a high income as the reference in the comparisons.

### Statistical analyses

Our stratified analysis provided a description of the prevalence of the dispensation of antibiotics across the 240 strata. We measured the associations between dispensation and the explanatory variables through prevalence ratios (PRs) obtained by Cox proportional hazards regressions with a constant follow-up time equal to 1 [20]. We calculated 99% confidence intervals (CIs) rather than 95% CIs to minimise the problem of multiple comparisons. We developed five consecutive Cox regression models. Model 1 included only age. Model 2 added sex, to which model 3 added income, with model 4 adding country of birth. Finally, model 5 included the 240 intersectional strata.

We assessed the DA for each model by calculating the area under the receiver operating characteristic curve (AUC), with 95% CIs [17]. The AUC was computed by plotting the true-positive fraction (i.e. sensitivity) against the false-positive fraction (i.e. 1–specificity) for binary classification thresholds of the predicted probability of antibiotic dispensation, and it thereby measured the ability of the regression model to discriminate between individuals who received any antibiotics and those who did not. The value of the AUC ranges from 0.5 to 1, with 1 representing perfect discrimination and 0.5 indicating no predictive accuracy. Using the criteria proposed by Hosmer and Lemeshow [21], we classified DA as absent or very weak (AUC= 0.5–0.6), weak (AUC >0.6–⩽0.7), strong (AUC >0.7– ⩽0.8) or very strong (AUC >0.8).

The incremental change in the AUC value (ΔAUC) between the models was also calculated in order to assess the improvements in DA obtained by a model compared to the previous one [14]. If any statistical interaction of effects was present in the intersectional variable, the AUC of model 5 would take a higher value than that of model 4.

IBM SPSS Statistics for Windows v22 (IBM Corp., Armonk, NY) was used to perform the statistical analyses.

## Results

The overall period prevalence of antibiotic dispensation during 2016–2017 was 29.9%. As seen in Tables I and II, antibiotic dispensation was more common among women than it was among men (PR=1.42 (1.42–1.43)). Furthermore, antibiotic dispensation was slightly more common among high income earners compared to those with a low income (PR=0.97 (0.97–0.97)). A small income gradient was present among men, among people born in Europe (excluding Nordic countries) and the USA, Canada or Australia, and in the oldest age groups (see Table I). While no substantial average differences could be seen with regards to country of birth, the lowest PR pertained to those born in the USA, Canada or Australia (PR=0.93 (0.90–0.97)). Higher PRs could also be seen in the older age groups (⩾75 years: PR=1.35 (1.34–1.36)), while antibiotic dispensation was least common in the youngest age group (PR=0.76 (0.54–0.77)).

**Table I.** Prevalence of antibiotic dispensation in Sweden during 2016–2017 in income groups, according to age, sex and country of birth.

		Antibiotics % (*n*)		
		Low income	Medium income	High income
Age (years)	7–14	20.2 (115,777)	21.1 (110,866)	21.2 (72,106)
	15–24	27.6 (582,198)	28.8 (348,730)	29.8 (155,348)
	25–34	27.9 (545,851)	26.4 (443,676)	26.1 (152,555)
	35–44	28.8 (296,866)	27.2 (429,190)	26.0 (432,875)
	45–54	29.3 (394,751)	27.0 (431,285)	25.7 (425,807)
	55–64	31.0 (251,527)	31.5 (370,566)	31.9 (492,666)
	65–74	33.2 (125,093)	34.6 (280,757)	35.6 (694,624)
	⩾75	34.7 (245,013)	36.6 (368,542)	39.2 (291,650)
Sex	Female	34.8 (1,372,473)	34.8 (1,441,977)	36.0 (1,244,236)
	Male	22.3 (1,184,603)	23.9 (1,341,635)	26.8 (1,473,395)
Country of birth	Sweden	29.0 (1,911,197)	29.4 (2,503,427)	30.9 (2,498,311)
	Nordic	30.7 (68,031)	33.2 (74,797)	33.0 (80,833)
	Europe	27.5 (174,623)	31.1 (92,034)	32.7 (78,153)
	USA, Canada, Australia	23.6 (7,397)	28.5 (4,755)	29.1 (5,683)
	Asia, Africa, Central and South America	29.5 (395,828)	30.0 (108,599)	29.4 (54,651)

**Table II.** Prevalence ratios (PR), area under the receiver operating characteristic curve (AUC) and the incremental change in the AUC value (ΔAUC) between the models compared to model 1.

	Model 1	Model 2	Model 3	Model 4
*Age (years)*				
7–14	0.76 (0.54–0.77)	0.76 (0.76–0.77)	0.77 (0.76–0.78)	0.77 (0.76–0.77)
15–24	1.04 (1.03–1004)	1.04 (1.03–1.05)	1.05 (1.04–1.05)	1.05 (1.04–1.06)
25–34	0.99 (0.99–1.00)	0.99 (0.99–1.00)	1.00 (0.99–1.01)	1.00 (0.99–1.01)
35–44	1.00 (0.99–1.00)	1.00 (0.99–1.00)	1.00 (0.99–1.00)	0.99 (0.989–1.00)
45–54	Ref.	Ref.	Ref.	Ref.
55–64	1.16 (1.15–1.17)	1.16 (1.15–1.16)	1.15 (1.14–1.16)	1.15 (1.15–1.16)
65–74	1.29 (1.28–1.29)	1.28 (1.27–1.29)	1.27 (1.26–1.28)	1.27 (1.26–1.28)
⩾75	1.35 (1.34–1.36)	1.31 (1.31–1.32)	1.31 (1.31–1.32)	1.32 (1.31–1.33)
*Sex*				
Female		1.42 (1.42–1.43)	1.43 (1.42–1.43)	1.43 (1.42–1.43)
Male		Ref.	Ref.	Ref.
*Income*				
Low			0.97 (0.97–0.97)	0.96 (0.96–0.97)
Middle			0.97 (0.96–0.97)	0.97 (0.96–0.97)
High			Ref.	Ref.
*Country of birth*				
Sweden				Ref.
Nordic				0.99 (0.98–1.00)
Europe				1.00 (0.98–1.00)
USA, Canada, Australia				0.93 (0.90–0.97)
Asia, Africa, Central and South America				1.05 (1.05–1.06)
*AUC*	0.55 (0.55–0.55)	0.59 (0.59–0.59)	0.59 (0.59–0.59)	0.59 (0.59–0.59)
*ΔAUC*		+0.04	+0.04	+0.04

Values are point estimations and 99% confidence intervals (CI) obtained from Cox regression modelling antibiotic prescription in relation to age, sex, income and country of birth.

The DA of the models was absent or very weak, ranging from AUC=0.55 for model 1 to AUC=0.59 for model 4. The ΔAUC from model 1 to model 2 was very small (+0.04), and was absent from model 2 to models 3 and 4. Thus, sex slightly increased the DA based only on age, while income and country of birth did not.

**Figure 1.**
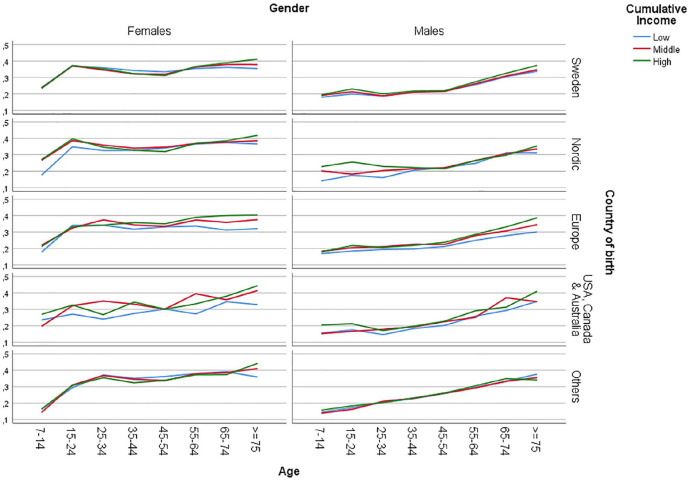
Prevalence of antibiotic dispensation in Sweden during 2016–2017, all intersectional strata.

The analysis of the intersectional variables revealed further heterogeneity (see Table III and Figure 1). Among the 10 groups with the lowest PRs, compared to the reference stratum (i.e. 45- to 54-year-old men with a high income born in Sweden), the majority (nine groups) were characterised by male sex, low income (five groups) and non-Swedish country of birth (10 groups). Of the latter, several were of Asian, African or South or Central American origin (five groups). Furthermore, all 10 strata belonged to the three youngest groups. The stratum with the lowest PRs consisted of males aged 7–14 years, with a medium income, born in South or Central America, Asia or Africa (PR=0.63 (0.47–0.86)).

**Table III.** Results from model 5, including the intersectional categorical variable.

Age (years)								Sex		Income			Country of birth					PR (99% CI)
7–14	15–24	25–34	35–44	45–54	55–64	65–74	⩾75	F	M	Low	Mid	High	Swe	Nord	Eur	USA, Canada, Australia	Africa, Asia, Central and South America	
																		0.63 (0.47–0.86)
																		0.64 (0.48–0.87)
																		0.66 (0.53–0.82)
																		0.66 (0.6–0.73)
																		0.67 (0.53–0.85)
																		0.70 (0.32–1.52)
																		0.71 (0.41–1.25)
																		0.72 (0.54–0.95)
																		0.73 (0.64–0.84)
																		0.74 (0.65–0.84)
																		1.81 (1.45–2.24)
																		1.83 (1.75–1.91)
																		1.84 (1.74–1.95)
																		1.87 (1.47–2.39)
																		1.87 (1.71–2.06)
																		1.88 (1.85–1.91)
																		1.89 (1.54–2.33)
																		1.91 (1.81–2.01)
																		2.02 (1.68–2.43)
																		2.03 (1.61–2.55)
*AUC*	0.60 (0.60-0-60)																	
*ΔAUC*	+0.05.																	

Values are PR with 99% CI for the 10 intersectional strata with the highest and lowest PR for antibiotic dispensation, compared with the reference stratum (i.e. 45- to 54-year-old men born in Sweden with a high income). The table also presents the value of the AUC with 95% CI and the ΔAUC compared to model 1 (Table II). Only the 10 intersectional strata with the highest and lowest PRs are shown.

The stratum with the highest PRs, compared to the reference stratum, was comprised of women aged ⩾75 years with a high income born in the USA, Canada or Australia (PR=2.03 (1.61–2.55)). Among the 10 groups with the highest PRs, all strata belonged to the oldest age groups, and most were female (nine groups). All groups had a high (seven groups) or medium (three groups) income. The majority originated from a country outside of Sweden and the other Nordic countries (eight groups).

The DA of the final model remained weak, with an AUC of 0.60. The ΔAUC from model 4 to model 5 was minute (+0.01). Thus, no considerable statistical interaction effects were observed.

## Discussion

This register study of antibiotic dispensation in Sweden in 2016–2017 shows that while dispensation was more common in older age groups and among women, no substantial differences pertained to country of birth. Although no large average differences were present with regards to income, the highest PR pertained to those with a high income compared to those with a low or medium income. Overall, the average differences were quite small, and the DA of the regression models was very low. This indicates small systematic differences in antibiotic dispensation associated with the variables under study due to large individual heterogeneity.

These results are partially in line with other studies. Women are known to be prescribed more antibiotics than men in Sweden [6,22], as are people in older age groups [2]. This can largely be explained by a higher prevalence of lower urinary tract infection in women than in men [23] and by a higher prevalence of co-morbidities with associated risks of infections among elderly people [6]. Meanwhile, the slightly more common antibiotic dispensations among those with a high income corroborates the conclusion of Hjern et al. [7] that children of highly educated parents in Sweden received more antibiotics in 1996–1997. However, Ternhag et al. [6] found that people with a low level of education received more antibiotics than those with a high level of education in 2010, while income had no linear effects on the dispensation. In another study, Melander et al. [24] found that children of parents with a high level of education received more antibiotics than those with a low level of education in southern Sweden, while the relationship was reversed in Denmark. Furthermore, Ternhag et al. [6] showed antibiotic dispensation to be more common among people born in Sweden than those born in other countries. In sum, and as noted in the introduction, research findings on the influence of socio-economic position diverge somewhat, as do those on the effect of country of birth.

While it is possible that the differences between the findings of our study and that of Ternhag et al. [6] mirror changes in prescription patterns between 2010 and 2016–2017, they may also be due to methodological issues. The study population of Ternhag et al. [9] included children younger than seven years of age, which is a group that consumes a considerable share of the prescribed antibiotics, while the present study did not. That said, the use of antibiotics among children aged 0–4 years has decreased since 2010 in Sweden [2]. Also, Ternhag et al. [6] compared those who had been prescribed antibiotics with a selected control population, while our study was based on the nationwide population. In any case, the diverging results with regards to socio-economic position, alongside our finding that antibiotic dispensation was on average slightly more common among those with a high income, is interesting in relation to AMR intervention strategies emphasising information campaigns [25]. Provided that a link between high income and high education can be assumed, our results counter the argument that a better-informed population group will necessarily consume fewer antibiotics.

A main strength of this study lies in the large nationwide database on which it was based. Nevertheless, we can only draw conclusions about correlations and not about causal relationships or underlying mechanisms behind the observed differences. Moreover, the data did not allow us to tie dispensation to the diagnoses motivating prescription, or to any existing co-morbidities. Our data did not include antibiotics dispensed from hospital wards or nursing home stockpiles, and as elderly people are more likely than younger ones to receive antibiotics from these locations, dispensation rates likely underestimate the use of antibiotics in older age groups. Furthermore, our information reflects dispensation rather than prescription or actual use of antibiotics. Socio-economic factors may affect the dispensation of prescriptions, and this may have had some impact on the result. Such effects are likely to have been at least partially ameliorated by the provision of medications free of charge to people <18 years of age in Sweden.

As for further limitations, it should be noted all that people in this study, including those born in another country, had been living in Sweden since at least 2010. Also, although children and elderly people account for a substantive share of all dispensed antibiotics, this study did not include children younger than seven years of age. The individualised disposable family income measure did not account for any changes in family composition or income during 2011–2017. Further, the variables used in this study can be seen as quite simplistic. For example, country of birth provides a blunt proxy for issues related to racialisation and migration [12]. Finally, with regard to the use of an intersectional approach, some researchers have questioned the compatibility of quantitative methods with intersectionality research [12], which has typically been qualitatively and theoretically oriented. However, others have argued for the importance of developing intersectional approaches in quantitative public-health research [12–14].

As noted in the introduction, and in response to calls for integration of intersectionality theory in social epidemiology and public health [12–14], potential contributions of intersectional AIHDA to research on health inequalities include increased specificity in the mapping of disparities, and information about the variability within and overlaps between social strata, in relation to the health outcome at hand. In this study, the mapping of disparities and of variability identified no large differences in antibiotic dispensation between socio-economic and demographic groups in Sweden for 2016–2017. Thus, and in accordance with the principle of proportionate universalism [26], public-health intervention aiming to support optimised prescription of antibiotics should be aimed towards the whole population. Similarly, our results suggest that in the Swedish context, increased attention on specific socio-economic or demographic groups appears to be less warranted in interventions aimed at improving prescription patterns among medical practitioners than the focus on optimisation through providing the proper diagnosis and prescription at the right time. While dispensation was indeed higher in some intersectional strata, the low DA indicates that interventions focused only on these would miss many individuals who are prone to antibiotic use but belong to strata with a lower prevalence; that is, because of the low DA, a focus on particular groups would yield many false-negatives (as well as false-positives). Furthermore, with regards to focus on particular groups, our study, like that of Ternhag et al. [6], speaks against tendencies in public debate towards attributing responsibility for infectious disease, irresponsible use of antibiotics and AMR to foreign-born or less educated population groups [9,10].

Heterogeneity in antibiotic dispensation can be explained by non-socio-economic factors, including differences in prescription habits among health-care centres and physicians [27–29] and varying degrees of concern about infectious illness among patients [30]. Further studies of socio-economic and non-socio-economic factors, and of their potential interactions, should distinguish between one-time or repeated use of antibiotics and include diagnoses and co-morbidities motivating prescription in the interest of furthering our understanding of patterns of antibiotic prescription and use.

## Conclusion

This study found small differences in antibiotic dispensation between socio-economic and demographic groups in Sweden. These results support universal public-health interventions and efforts towards improving prescription patterns among medical practitioners aiming to support the reduced and optimised use of antibiotics overall, rather than targeting specific population groups.
